# Reversing type 1 diabetes with stem cell–derived islets: a step closer to the dream?

**DOI:** 10.1172/JCI158305

**Published:** 2022-02-01

**Authors:** Peter C. Butler, Edwin A.M. Gale

**Affiliations:** 1 Larry Hillblom Islet Research Center, Department of Medicine, UCLA, Los Angeles, California, USA.; 2 Diabetes and Metabolism, University of Bristol, Bristol, United Kingdom.

Ernest McCulloch and James Till established the properties of stem cells in the 1960s (1), fueling interest in exploiting the ability of pluripotent cells to replace dysfunctional tissue in disease states. This concept has particular appeal for type 1 diabetes, a condition in which the loss of approximately 0.8 g of pancreatic β cells results in a lifelong need for insulin injections. Added to this, type 1 diabetes can be reversed by a pancreas or islet cell transplant, which provides proof of principle for cell-based therapy (2). Whole pancreas transplants require major surgery, and 99% of the allograft has no relevance to insulin secretion. Intraportal infusion of islets isolated from the pancreas of brain-dead donors has been carried out with partial success for more than 20 years. Barriers to wider use of isolated islets include a marked shortage of suitable donors, physical and ischemic damage to islets during isolation and transfer, and the need for long-term immunosuppression. Even so, insulin independence can be transformative for those who achieve it.

## Recent progress in cell-based therapies for diabetes

Stem cell–derived insulin-producing cells can theoretically be generated in endless quantities, potentially overcoming the constraints in both supply and viability. Two companies, ViaCyte and Vertex, have recently reported preliminary data from ongoing clinical trials of stem cell–based therapies for type 1 diabetes. The approaches are very different. Vertex embarked on a phase I/II clinical trial in March 2021 in which a proprietary embryonic stem cell–derived (ESC-derived) therapy is delivered into the portal circulation by infusion under immunosuppressive coverage. Vertex transplants ESC-derived islet cells that include functional β cells using a differentiation protocol established by Melton and colleagues at Harvard University (3). The FDA required that the first patients receive no more than 50% of the planned replacement dose. Despite this limitation, the 90-day results for the first patient to enter the trial showed an increase in fasting and meal-stimulated C-peptide levels from undetectable to 280 and 560 pmol/L, respectively, in association with a reduction in HbA1c levels and a decrease in the required therapeutic insulin dose from 34 to 3 units or less daily (4). This encouraging result, promulgated by a press release on October 18, 2021, has been widely reported in the media, raising the hopes and expectations of individuals with type 1 diabetes and their families.

ViaCyte (formerly Novocell and BetaLogics) launched its first clinical trial in March 2014 with a device named VC-01, which encapsulates pancreatic endoderm prepared according to a proprietary differentiation protocol developed by the company. This strategy is based on the principle, defined in preclinical studies, that the pancreatic endoderm will complete its differentiation to functional islet cells after transplantation. The ESC-derived endoderm was enclosed within a protective membrane designed to prevent a host immune response, but trial of a modified device called VC01-103 was terminated due to “insufficient functional product engraftment,” which suggests that the recovered devices were coated with fibrous tissue (5, 6). In a subsequent trial, portals that permitted vascular ingrowth were added to the device, but these also conferred access to the immune system, which made induction and maintenance immunotherapy necessary. Recently, Shapiro and colleagues reported findings from a multicenter trial that included 17 patients (7), and Ramzy and colleagues reported on a single-center study of 15 individuals that focused on clinical measures including sequential meal studies (8).

In contrast to the sealed capsule, the implanted “open” device resulted in survival of endoderm-derived cells in some of the patients. Shapiro et al. reported that 6 of the 17 patients, each of whom received subcutaneous devices with a net capacity of 1 million islet equivalents, developed detectable glucose-stimulated C-peptide (range, 33–99 pmol) during the 12–24 months of study, whereas 11 of 17 had no detectable C-peptide. However, insulin production did not reach therapeutic levels, and there was no discernible clinical benefit in those who achieved detectable C-peptide levels. This outcome is not surprising, as even grafts from responders displayed extensive fibrosis, and host fibroblasts were the most abundant cell type in the devices. Another potential problem with a therapy based on pancreatic endoderm is that the trajectory of differentiation is variable and unpredictable. This issue might explain the abundance of glucagon-expressing cells, whereas insulin-expressing cells — when present — were a small minority in the grafts.

Ramzy et al., meanwhile, assessed graft-related insulin secretion with a highly sensitive ELISA method for measurement of C-peptide levels. This assay has a limit of detection of 0.017 pmol/L, which made it possible to examine levels of endogenous insulin secretion in those in whom C-peptide secretion was undetectable by the standard method (9). These very low levels of C-peptide increased with time after implantation of devices, especially in older individuals. C-peptide is cleared by the kidney, and immunosuppression, as achieved in the study, can impair renal function, especially in older people (10). Decreased clearance of C-peptide — potentially derived from residual pancreatic β cells rather than from the implanted devices — might therefore have contributed to the observed increase. Clinical measures such as decreased insulin requirement or time spent within the target blood glucose range did not correlate with C-peptide level or with the number of insulin-positive cells detected in the grafts. Clinical improvement is well documented in trials of new therapies for diabetes, regardless of the nature of the intervention (11). Patient 11, for example, showed the greatest reduction in insulin requirement during the trial but also experienced the greatest weight loss, which would be predicted to enhance insulin sensitivity; he was withdrawn from the study as a C-peptide nonresponder at 9 months. The report’s summary does not mention that the study did not achieve therapeutic levels of insulin secretion or provide unequivocal evidence of clinical benefit. Even if the devices were truly responsible for the reported increment in C-peptide, our understanding is that clinical impact has yet to be demonstrated and that an impossibly large number of implanted devices would be required to achieve it.

## Cautious interpretation and barriers to overcome

These studies speak to the urgent hopes and dreams of millions of people with type 1 diabetes, but it is far too early to break out the champagne. The dramatic result from the first patient in the Vertex trial, if reproduced, might come to be seen as the first clear demonstration that differentiation of stem cells into islet-like structures containing glucose-responsive insulin-secreting cells can reverse diabetes in humans. If so, this might come to be seen as a landmark achievement, akin to the first use of insulin injection for treatment of diabetes in a person in 1922. Could this be a Leonard Thompson moment in the history of diabetes? As things stand, the need for long-term immunosuppression may limit clinical application of both the ViaCyte and Vertex approaches; this is clearly a risk only worth taking if diabetes can be fully reversed for a sustained period.

The alternatives to immunosuppression are to establish immune tolerance toward the transplanted cells or to create a “privileged site” for the transplant. Both companies are wrestling with this dilemma. ViaCyte is to be commended for its attempts to encapsulate cells in a protective membrane that bars access to immunocytes, but it seems clear that the first-generation devices did not work in humans because they were coated in fibrous tissue. The subsequent resort to devices that permit vascular access — and make immunosuppression necessary — might be taken as an admission of failure. Furthermore, host responses to the open devices have included invasion by fibroblasts and acellular material, and the demonstration that insulin-producing cells can survive within such devices still falls short of a realistic replacement therapy for type 1 diabetes. The companies also diverge in their approach to differentiation. ViaCyte has pinned its hopes on undirected differentiation from pancreatic endoderm to islet endocrine tissue, whereas Vertex has opted to take the cells further along the directed differentiation pathway, which might explain the marked difference in outcome to date.

It is important to note that the Vertex result is currently based on a single patient; and that ViaCyte’s strategy, based on differentiation of pancreatic endoderm to sufficient numbers of functional β cells within porous capsules, has not as yet been shown to work. More information is eagerly awaited on what promise to be transformational therapeutic options for people with type 1 diabetes.

## Version 1 02/01/2022

Electronic publication
